# A Polymorphism in *RNF213* Is a Susceptibility Gene for Intracranial Atherosclerosis

**DOI:** 10.1371/journal.pone.0156607

**Published:** 2016-06-02

**Authors:** Oh Young Bang, Jong-Won Chung, Jihoon Cha, Mi Ji Lee, Je Young Yeon, Chang-Seok Ki, Pyoung Jeon, Jong-Soo Kim, Seung Chyul Hong

**Affiliations:** 1 Department of Neurology, Samsung Medical Center, Sungkyunkwan University School of Medicine, Seoul, Republic of Korea; 2 Department of Radiology, Samsung Medical Center, Sungkyunkwan University School of Medicine, Seoul, Republic of Korea; 3 Department of Neurosurgery, Samsung Medical Center, Sungkyunkwan University School of Medicine, Seoul, Republic of Korea; 4 Department of Laboratory Medicine and Genetics, Samsung Medical Center, Sungkyunkwan University School of Medicine, Seoul, Republic of Korea; Temple University School of Medicine, UNITED STATES

## Abstract

**Background:**

Both intracranial atherosclerotic stenosis (ICAS) and moyamoya disease (MMD) are prevalent in Asians. We hypothesized that the Ring Finger protein 213 gene polymorphism (*RNF213*), a susceptibility locus for MMD in East Asians, is also a susceptibility gene for ICAS in patients whose diagnosis had been confirmed by conventional angiography (absence of basal collaterals) and high-resolution MRI (HR-MRI, presence of plaque).

**Methods:**

We analyzed 532 consecutive patients with ischemic events in the middle cerebral artery (MCA) distribution and relevant stenotic lesion on the distal internal carotid artery or proximal MCA, but no demonstrable carotid or cardiac embolism sources. Additional angiography was performed on 370 (69.5%) patients and HR-MRI on 283 (53.2%) patients.

**Results:**

Based on angiographic and HR-MRI findings, 234 patients were diagnosed with ICAS and 288 with MMD. The *RNF213* variant was observed in 50 (21.4%) ICAS patients and in 119 (69.1%) MMD patients. The variant was observed in 25.2% of patients with HR-MRI-confirmed ICAS. Similarly, 15.8% of ICAS patients in whom MMD was excluded by angiography had this variant. Among the ICAS patients, *RNF213* variant carriers were younger and more likely to have a family history of MMD than non-carriers were. Multivariate testing showed that only the age of ICAS onset was independently associated with the *RNF213* variant (odds ratio, 0.97; 95% CI, 0.944–0.99).

**Conclusions:**

*RNF213* is a susceptibility gene not only for MMD but also for ICAS in East Asians. Further studies are needed on *RNF213* variants in ICAS patients outside East Asian populations.

## Introduction

Intracranial atherosclerotic stenosis (ICAS) is a common stroke subtype worldwide. Although ICAS is more prevalent in Asians than in Westerners, the reason for the racial-ethnic differences is unknown. Possible explanations include inherited susceptibility to intracranial vessel atherosclerosis,[1] acquired differences in risk factor prevalence,[2,3] and differential responses to the same risk factors.[4–6]

Moyamoya disease (MMD) is an idiopathic intracranial arterial disease characterized by progressive stenosis of the distal internal carotid artery (ICA) and a hazy network of basal collaterals called moyamoya vessels. The main pathological changes of the stenotic segment in MMD are the fibrocelluar thickening of the intima, irregular undulation of the internal elastic laminae, medial thinness, and a decrease in the outer diameter, while focal thickness of intima due to atheroma and subintimal hemorrhage is the main feature of ICAS.

Although much progress has been made in our understanding of MMD,[7] the etiology of MMD is unknown, and no medication can stop or reverse its progression. At present, surgical re-vascularization is the mainstay MMD treatment, On the contrary, vascular risk factor control, aggressive medical management (including statins), and stent placement (in selected patients) are important for preventing stroke in patients with ICAS. A genome-wide linkage analysis and exome analysis recently identified the ring finger protein 213 gene (*RNF213*) on 17q25.3 as the strongest susceptibility gene for MMD in East Asian people.[8,9] However, this genetic variant associated with MMD was also observed in patients with non-MMD intracranial stenosis.[10,11] [12]

We hypothesized that the increased prevalence of ICAS and MMD in East Asians may in part be caused by a common genetic background. Specifically, the genetic factor associated with MMD may be a susceptibility gene in ICAS. Thus, we tested *RNF213* variants in patients whose diagnosis of ICAS had been confirmed by high-resolution magnetic resonance imaging (HR-MRI; the presence of plaque) and conventional angiography (the absence of basal collaterals). In addition, the clinicoradiological characteristics of patients with ICAS depending on the presence or absence of this variant were analyzed.

## Materials and Methods

From January 2008 to April 2015, patients with ischemic cerebrovascular events in the middle cerebral artery (MCA) distribution, who were admitted to a University Medical Center, were prospectively recruited. Potential participants were defined as patients experiencing focal or lateralizing symptoms within the MCA distribution and showing ≥50% stenosis or occlusion at terminal portions of the ICA and/or proximal MCA on conventional or MR angiography. Based on the Stop Stroke Study Trial of Org 10172 in Acute Stroke Treatment (SSS-TOAST), patients with potential sources of cardioaortic embolism, extracranial atherosclerosis with significant (≥50%) stenosis on the relevant extracranial arteries, other stroke mechanisms (coagulopathy, vasculitis, arterial dissection, etc.), or incomplete evaluations were excluded. Local institutional review board (Samsung Medical Center Institutional Review Board) approved this study. One hundred thirty-seven patients with other stroke subtypes (e.g., lacunar stroke or cardioembolism) and 83 healthy control subjects served as the control group. All patients or patient guardians provided written informed consent for participation in this study.

Clinical information, including age, gender, and vascular risk factors, was collected. All patients underwent standardized diagnostic tests that included routine blood tests (total cholesterol, triglyceride, high-density and low-density lipoprotein-cholesterol, C-reactive protein, etc.) and cardiac work-ups (electrocardiography, ≥24-h cardiac telemetry or echocardiography).

A diagnosis of intracranial stenosis was made on the basis of conventional angiography or magnetic resonance angiography (MRA). Conventional angiography was performed, especially when the presence of basal collaterals was highly suspicious, when vascular stenosis progression was observed, or when a neurointervention such as stenting was considered. Patients underwent comprehensive diagnostic cerebral angiography, including injection of internal and external carotid arteries and the dominant vertebral artery.

HR-MRI was performed on patients, especially when vascular studies gave controversial results in the diagnosis of MMD or ICAS or when conventional angiography was unable to be performed. Details of HR-MRI parameters are described in our previous work.[13] HR-MR images were analyzed to evaluate the vessel walls. We evaluated the MCA or basilar artery (BA) at the site of maximal stenosis or just proximal to the occluded site on 3D time-of-flight MRA. Normal vessels that were located contralateral or proximal to the stenotic portion were also assessed for reference values. The lumen and the outer vessel contours were manually traced on T2/proton density-weighted images using an image-analyzing program (Medical Image Processing Analysis and Visualization [MIPAV]; Center for Information Technology, National Institutes of Health, Bethesda, MD, USA). The lumen area (LA) and vessel area (VA) were automatically calculated after tracing by the program. The wall area (WA) was defined as the difference between the VA and LA (i.e., WA = VA–LA). The degree of stenosis was calculated as follows: (1 –LA of MCA or BA/reference LA) × 100%. The remodeling index (RI) was the ratio of VA at the MCA or BA to the reference vessel. The pre- and post-contrast T1 fluid-attenuated inversion recovery (FLAIR) images were compared to determine the presence or absence and the pattern of enhancement. Enhancement was considered concentric if it was uniform or circumferential. It was regarded as eccentric if it was not 360° circumferential or if the thickest part was more than twice the thinnest part, where circumferential enhancement was observed. Two neurologists (M.J.L. and J.W.C) read the HR-MR images. In cases of disagreement, a third reader was invited to resolve the issue. The enhanced area (EA) was also manually drawn. All quantitative data were re-measured 2 weeks later by a neurologist (J.W.J.) to estimate intra-observer variability.

Genomic DNA was extracted from peripheral blood leukocytes using a Wizard Genomic DNA Purification kit and following the manufacturer’s instructions (Promega, Madison, WI, USA). The c.14429G>A (p.Arg4810Lys) mutation of the *RNF213* gene (GenBank accession number NM_001256071.1) was amplified using primer sets designed by the authors (available upon request). A polymerase chain reaction was performed with a thermal cycler (model 9700; Applied Biosystems, Foster City, CA, USA), and direct sequencing was performed with a BigDye Terminator Cycle Sequencing Ready Reaction kit (Applied Biosystems) on an ABI Prism 3730*xl* genetic analyzer (Applied Biosystems).

Commercially available software (SPSS, version 18.0; SPSS Inc., Chicago, IL, USA) was used for the statistical analyses. Differences in discrete variables among the groups were examined via χ^2^, Fisher exact, and Mann-Whitney tests. Differences in continuous variables were examined using 1-way analyses of variance, Kruskal-Wallis tests, and *t* tests. In addition, independent factors for *RNF213* mutation were evaluated using logistic regression. Univariate analyses of variables with *P <* 0.2 were considered explanatory variables and were evaluated together in subsequent multivariate analyses. *P <* 0.05 was considered statistically significant.

## Results

**Fig 1:**
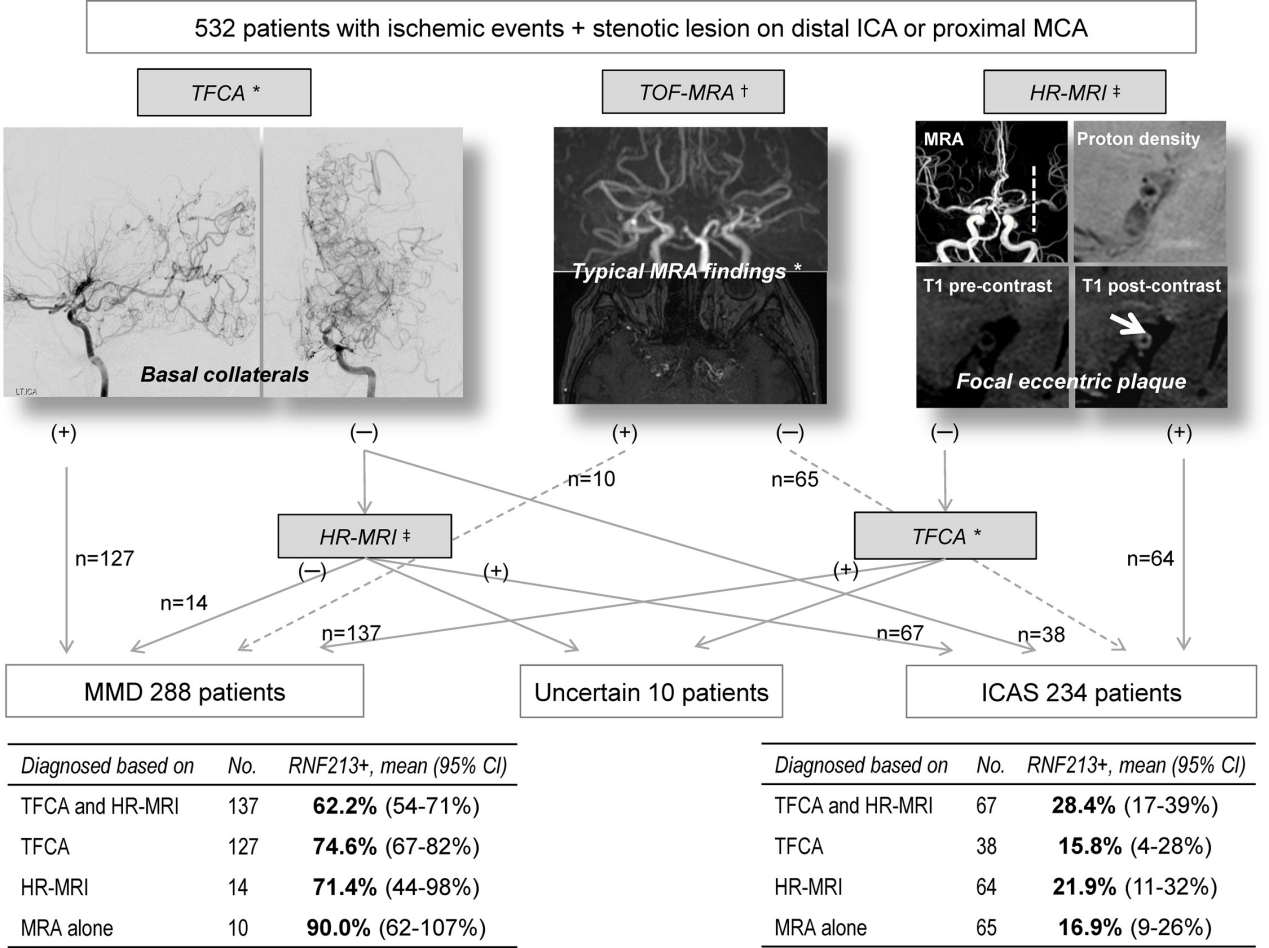
Patient groups and the prevalence of the RNF213 variant. * Transfemoral cerebral angiography (TFCA) finding of moyamoya disease (MMD) indicate the presence of basal collaterals. †Time-of-flight magnetic resonance angiography (TOF-MRA) findings of MMD indicate bilateral appearance of an abnormal vascular network in the basal ganglia on the source image of MRA. ‡High-resolution magnetic resonance imaging (HR-MRI) findings of intracranial atherosclerotic stenosis indicate the absence of focal eccentric plaque and presence of negative remodeling and concentric enhancement. Abbreviations: ICA, internal carotid artery; MCA, middle cerebral artery, TFCA, transfemoral cerebral angiography; HR-MRI, high-resolution magnetic resonance imaging; MRA, magnetic resonance angiography; MMD, moyamoya disease; ICAS, intracranial atherosclerotic stroke.

Of 532 patients with intracranial stenosis, 317 (59.6%) were female and the average age was 50.4 ± 13.4 years (range: 22–93 years). Additional conventional angiography was performed on 370 (69.5%) patients and HR-MRI on 283 (53.2%) patients. Based on the conventional angiography and HR-MRI findings, 234 patients were diagnosed with ICAS, and 288 with MMD (Fig 1). Ten patients for whom the diagnosis was uncertain and who showed mismatching results between conventional angiography and HR-MRI findings were excluded from this study. Steno-occlusive lesions on the terminal ICA were observed in 311 patients, and the remaining 211 patients had proximal MCA lesions without terminal ICA lesions. Characteristics of the patients with ICAS and MMD are shown in Table 1.

**Table 1:** Characteristics of patients.

	MMD (n = 288)	ICAS (n = 234)	*P*-value
Age at diagnosis, mean (SD)	45.9 (12.7)	56.0 (12.2)	<0.001
Vascular risk factor, n (%)			
Male gender	87 (30.2)	120 (51.3)	<0.001
Hypertension	129 (44.8)	135 (57.7)	0.002
Diabetes	34 (11.8)	66 (28.2)	<0.001
Hyperlipidemia	78 (27.1)	113 (48.3)	<0.001
Family history of MMD, n (%)	29 (10.1)	5 (2.1)	<0.001
*RNF213* mutation, n (%)	199 (69.1)	50 (21.4)	<0.001

MMD, moyamoya disease

The *RNF213* variant was observed in 21.4% (50 of 234) of patients with ICAS as well as 69.1% (199 of 288) of patients with MMD, but rarely in non-MMD, non-ICAS patients (3.6%, 5 of 137 patients) and healthy control patients (1.2%, 1 of 83 patients). Non-MMD, non-ICAS patients included lacunar stroke in 46, cardioembolism in 46, carotid atherosclerotic stroke in 9, and other etiologies (such as arterial dissection and vasculitis) in 36 patients. A substantial proportion of patients (28.4%, 19 of 67 patients) in whom ICAS was confirmed by the presence of focal eccentric plaque on HR-MRI and absence of basal collaterals (moyamoya vessels) on conventional angiography had the *RNF213* variant (Fig 1); 25.2% (33 of 131 patients) of the ICAS group who had focal eccentric plaques without negative remodeling on the relevant stenotic segment on HR-MRI had this variant. Similarly, 15.8% (6 of 38) of patients with ICAS in whom MMD was excluded by conventional angiography had this variant.

Table 2 shows characteristics of the patients with ICAS depending on the presence of the *RNF213* variant. Among these patients, *RNF213* variant carriers were younger (52.6 ± 9.6 vs. 56.9 ± 12.7 years, *P* = 0.027) and more likely than non-carriers to have a family history of MMD (6.0% vs. 1.1%, *P* = 0.033). There were no differences among the patients regarding other clinical characteristics, including vascular risk factors, MRA findings (the site of stenosis or presence of tandem lesions) and HR-MRI findings. Multivariate testing was performed to further evaluate factors associated with the presence of *RNF213* among 234 patients of the ICAS group. Results showed that the age of ICAS onset was independently associated with the *RNF213* variant (odds ratio, 0.97; 95% CI, 0.944–0.99; *P* = 0.014).

**Table 2:** Factors associated with the presence of *RNF213* genetic variants among 234 patients with intracranial atherosclerotic stenosis.

	*RNF213*			Univariate		Multivariate	
	Present (n = 50)	Absent (n = 184)	*P*-value	OR (95% CI)	*P*-value	OR (95% CI)	*P*-value
Age at diagnosis	52.6 ± 9.6	56.9 ± 12.7	0.027	0.97 (0.94–0.99)	0.028	0.97 (0.94–0.99)	0.014
Vascular risk factor							
Male gender, n (%)	23 (46.0)	97 (52.7)	0.399	0.75 (0.41–1.43)	0.400	0.63 (0.33–1.22)	0.170
Hypertension	26 (52.0%)	109 (59.2%)	0.358	0.75 (0.40–1.40)	0.359		
Diabetes	11 (22.0%)	55 (29.9%)	0.272	0.66 (0.32–1.39)	0.274		
Hyperlipidemia	25 (50.0%)	88 (47.8%)	0.785	1.09 (0.58–2.04)	0.785		
Family history of MMD	3 (6.0%)	2 (1.1%)	0.033	5.81 (0.94–35.77)	0.058	4.98 (0.78–31.70)	0.089
Terminal ICA stenosis	16 (32.0%)	56 (30.4%)	0.832				
Tandem stenosis*							
Proximal carotid artery	5 (10.0%)	11 (6.0%)	0.344	1.75 (0.58–5.29)	0.323	2.64 (0.82–8.51)	0.104
Posterior circulation territory	4 (8.0%)	17 (9.2%)	1.000	0.85 (0.27–2.66)	0.786		
HR-MRI findings† (n = 128)	n = 32	n = 96					
Presence of enhancement	29 (90.6)	75 (78.1)	0.117				
Enhanced volume (mm^3^)	58.5 ± 52.15	56.4 ± 50.8	0.850				
Remodeling index	0.95 ± 0.19	0.93 ± 0.25	0.714				

MMD, moyamoya disease; ICA, internal carotid artery; HR-MRI, high-resolution magnetic resonance imaging.

*Asymptomatic side.

†Symptomatic vessels.

## Discussion

The major findings of this study are that (a) more than one fifth of patients confirmed by HR-MRI and conventional angiography to have ICAS had a genetic variant associated with MMD, and (b) patients with this variant had an earlier onset of ICAS than those without it.

No genetic factors specific to ICAS have been reported. Although several genetic factors were reportedly associated in patients with ICAS, such as polymorphisms in adipocytokines, lipoprotein lipase, and C-reactive protein, they were also associated with extracranial atherosclerosis.[14] In addition, recent genome-wide association studies have shown stroke subtype-sensitive genetic factors.[15] However, they merged intracranial and extracranial atherosclerosis for the study.

The *RNF213* genetic variant was identified in 95% of patients with familial MMD, in 80% of patients with sporadic MMD, and in 1.8% of control patients.[16] Recently, Miyawaki and colleagues suggested that a particular subset of Japanese patients with non-MMD intracranial stenosis had a genetic variant associated with MMD.[10,11] In their studies, 22–24% of “ICAS” patients had the *RNF213* variant. However, intracranial stenosis was diagnosed based on time-of-flight MRA, and vascular wall changes or basal collaterals were not evaluated in these studies. These patients may have MMD rather than ICAS, even though they did not have signs of MMD (e.g., basal collaterals). The angiography-based criteria have limitations in that they are based on childhood MMD data, and unlike childhood MMD, it is often difficult to differentiate ICAS from MMD in adult patients with intracranial arterial stenosis.[12] In some patients without basal collaterals, typical angiographic findings of MMD developed on follow-up angiography.[12] Therefore, in the present study, we performed HR-MRI and conventional angiography to exclude the ICAS-mimicking MMD cases. This is the first study evaluating the frequency of the RNF213 genetic variant in patients with HR-MRI documented intracranial atherosclerotic stenosis. HR-MRI may reveal characteristic vessel wall changes of MMD (shrinkage) rather than atherosclerotic plaque on the stenotic segment.[17,18] Our present study of HR-MRI- and conventional angiography-confirmed ICAS cases indicates that the *RNF213* variant is also associated with ICAS, and suggests that ICAS and MMD share common genetic polymorphisms. The population carrying the *RNF213* variant was estimated to be 16.16 million people in East Asian countries.[19] Results of the present study raise the possibility that this variant could contribute to the high prevalence of intracranial atherosclerotic stroke in Asians.

Although several studies have reported on the role of the *RNF213* gene in vascular development,[8,9] there are no studies on its role in atherosclerotic diseases. Possible explanation for the association between ICAS and the *RNF213* gene variant may include as followings. First, the *RNF213* gene is reportedly associated with vascular risk factors, such as blood pressure.[20] Second, caveolae are 50- to 100-nm cell surface plasma membrane invaginations that are abundant in endothelial cells, and caveolin-1, a scaffolding protein of the caveolae plasma membrane, is reportedly involved in the pathogenesis of cancers and vascular diseases.[21] Down-regulation of caveolin-1 reportedly reduced capillary formation *in vitro* and *in vivo*, and was associated with pathologic angiogenesis.[21–23] In our unpublished data, the caveolin-1 level was markedly decreased in patients with MMD, especially in patients with the *RNF213* variant, and to a lesser degree in those with ICAS. Further studies are needed to evaluate the impact and interactions between *RNF213* and the *CAV1* gene encoding caveolin-1.[24] Third, it is possible that certain non-*RNF213* genetic factors cause MMD and ICAS. Smooth muscle proliferation may be the key pathogenic mechanisms of occlusion in MMD, and one study of 20 families with a mutation in smooth muscle α-actin (ACTA2) showed that this mutation can cause a diversity of vascular diseases, including the premature onset of coronary artery disease and stroke, and MMD.[25]

In addition, our results showed that ICAS patients with the *RNF213* variant were younger than those without this variant. Patients with this variant may be prone to atherosclerosis.[26] The *RNF213* variant could lead to vascular fragility, which may render vessels more vulnerable to hemodynamic stress and secondary insults.[26] Besides vascular risk factors, various environmental factors (e.g., autoimmune response and infection/inflammation) are suggested to trigger vascular abnormalities in the presence of the *RNF213* variant. Elevated thyroid autoantibodies were associated with MMD[27–29] and recently with ICAS.[30] Serial HR-MRI studies are needed to evaluate the particular effects of these factors on the development and progression of plaque in the *RNF213* variant carriers. In the present study, we have investigated the vascular (tandem stenosis or distal ICA involvement) and HR-MRI (remodeling pattern and plaque enhancement for vulnerability of plaque) characteristics in ICAS patients with this variant, but failed to find significant differences depending on the presence or absence of this variant. This could be because the progression of atherosclerosis is possibly influenced by both vascular risk factors and genetic susceptibility.

This study had several limitations. First, the study was cross-sectional and had a limited sample size. A long-term follow-up study of a large cohort is needed. Second, conventional angiography and HR-MRI were not performed on all patients, but on selected patients. However, as shown in Fig 1, we used a systematic approach in the use of HR-MRI and conventional angiography to minimize the number of tests. Third, the participants in our study are not representative of the general population with intracranial stenosis, because this was a single-center study at a tertiary referral center where stenting for ICAS and bypass surgery for MMD are actively preformed. Moreover, the results of this study cannot be generalized outside of East Asians, because the *RNF213* variant is not the susceptibility gene for MMD in Westerners or South Asians. Several non-p.Arg4810Lys *RNF213* variants (rs148731719 and rs397514563) were recently found in Caucasian and East and South Asian cases with MMD.[9,31–33] In addition, clinical manifestations and possibly angiographic findings may differ between Westerners and East Asians.[34] The p.Arg4810Lys *RNF213* variant is reportedly related to ischemic-type MMD, whereas non-p.Arg4810Lys *RNF213* variants (especially A4399T) are associated with hemorrhagic-type MMD.[31] Further studies on biomarkers in these populations are needed.

## Conclusions

In conclusion, our data indicate that *RNF213* is a susceptibility gene not only for MMD but also for ICAS, which may in part explain the high prevalence of intracranial atherosclerotic stroke in Asians. We have an ongoing prospective follow-up study of patients with intracranial stenosis involving genetic, protein, and neuroimaging biomarkers (NCT02074111 at Clinical.trial@gov). Further studies are needed on the non-p.Arg4810Lys *RNF213* variants in ICAS patients who are not of East Asian descent.
